# Shea Nut Oil Extracts Enhance the Intra-Articular Sodium Hyaluronate Effectiveness on Surgically Induced OA Progression in Rats

**DOI:** 10.3390/nu12040957

**Published:** 2020-03-30

**Authors:** Ing-Jung Chen, Chih-Shung Wong

**Affiliations:** 1 Department of Anesthesiology, Cathay General Hospital, Taipei 10630, Taiwan; dr.jungchen@gmail.com; 2 Department of Medical Research, Cathay General Hospital, Taipei 10630, Taiwan; 3 Graduate Institute of Medical Sciences, National Defense Medical Center, Taipei 11490, Taiwan

**Keywords:** shea nut oil, lipids, triterpenes, osteoarthritis, pain, hyaluronic acid, triterpenes, cartilage

## Abstract

Osteoarthritis (OA) progression is associated with joint pain and stiffness. Intra-articular hyaluronic acid (IAHA) injection in knee OA restores the viscoelasticity of the joint and prevents cartilage damage. Shea nut oil extract (SNO) was shown to provide chondroprotection on surgically-induced OA progression in rats. Here we aim to examine IAHA injection supplemented with SNO diet for a synergetic evaluation on the disease progression in OA rats. We employed an anterior cruciate ligament transection plus medial meniscectomy-induced knee OA rat model with up to 12 weeks of sign/behavior observation (knee width, weight-bearing) and histological assessments of joint damage. We found both IAHA and SNO alone significantly attenuated histological changes of cartilage degeneration and synovial reactions in these knee OA rats. Nonetheless, oral SNO alone mitigated OA pain and inflammation while IAHA alone had no significant impact on the weight-bearing test and knee joint swelling. Moreover, with IAHA-treated rats fed with oral SNO diet, additional anti-inflammatory and anti-nociceptive effects were found, which further enhanced and maintained IAHA protection. Given the differential phenotype of oral SNO vs. IAHA, a regimen of IAHA coupled with SNO supplement provides a long-term effect of IAHA treatment. Taken together, the SNO supplement can be safely used as an adjuvant diet for chronic symptomatic relief of OA coupled with IAHA management.

## 1. Introduction

Osteoarthritis (OA) is a multifactorial joint disease and a common disabling condition affecting the global population [1,2]. The increasing joint pain and stiffness gradually leads to reduced physical function, quality of life, and frequent physician visits [3,4]. Up to 54.4 million adults experienced doctor-diagnosed arthritis during 2013–2015 in United States, and 23.7 million had arthritis-attributable activity limitations. In addition, adults with heart disease, diabetes, and obesity have a higher prevalence of OA (49.3%, 47.1%, and 30.6%, respectively) and consequently arthritis-attributable activity limitations [5]. Therefore, the management of these associated factors are recommended to potentially reduce symptomatic knee and hip OA incidences [6,7].

OA pain is the predominant limiting factor for a patient’s activity and life quality, and it leads those individuals affected to seek medical care [8]. The pain tends to be localized to the affected joint and aggravated by joint use while relieved by rest. The ultimate goal of nonsurgical treatment modalities is to reduce the pain and restore function while delaying total knee replacement (TKR), a substantial direct health-care cost in OA patients with end-stage disease. Researchers have urged into the preventive management of OA and development of disease-modifying OA drugs [9].

HA (hyaluronic acid) is an intrinsic component within the knee joint and provides viscoelastic properties to synovial fluid. Increasing HA levels through intra-articular (IA) injection restores the viscoelasticity of the synovial fluid, which aids shock absorption, lubrication, and protection of the joint, along with a good safety profile [10,11,12]. Moreover, several retrospective studies have shown the potential of IAHA (intra-articular hyaluronic acid) to delay the time for TKR in patients with OA [13,14,15,16].

Shea nut oil (SNO), extracted from the African shea tree (Vitellaria paradoxa), contains a high nutritional value with high triterpene and oleic/stearic fatty acid concentration, vitamins, and minerals. Importantly, their high triterpene alcohol and tocopherol content are considered to have anti-inflammatory and anti-oxidant properties [17]. Clinical evidence suggests the bioactive triterpene concentrate has anti-inflammatory effects under daily oral supplementation in OA patients [18]. Additionally, a clinical report demonstrated a decrease in pain and stiffness in patients after daily oral SNO supplement for 16 weeks [19]. We previously reported that preventive oral administration of SNO dose-dependently reduces cartilage degeneration in a rat model of anterior cruciate ligament transection plus medial meniscectomy (ACLT + MMx)-induced OA [20]. It also reduces pain and provides differential cartilage protection in both acute and chronic OA rats [21].

Regarding the complexity of this natural plant oil, the overall protective effect may be derived from a combined mechanism of actions of the different triterpene concentrates (primary α, β-amyrin, lupeol, and butyrospermol), monounsaturated oleic acid, or tocopherol found in this shea nut oil product. For instance, in mouse inflammation models induced by complete Freund’s adjuvant and by partial sciatic nerve ligation, daily oral intake of α and β-amyrin showed long-lasting antinociceptive and anti-inflammatory effects via direct activation of cannabinoid receptors and a concomitant inhibition of inflammatory NF-κB, cyclic adenosine monophosphate response element binding (CREB) pathway [22]. Otuki et al.’s report suggested that the antinociceptive properties of mixed amyrins may be involved in the inhibition of protein kinase A and protein kinase C pathways [23]. Others researchers found that lupeol acetate ameliorates collagen-induced arthritis through suppression of inflammatory cytokines and inhibition of bone erosion [24]. Indeed, the potential antioxidant and free radical scavenging effects of amyrin and lupeol have been demonstrated both in vitro and in vivo [25,26,27,28]. A recent report showed increased antioxidant activity and suppressed proinflammatory cytokine expressions in obese OA rats fed with SNO, further consolidating our previous findings [29]. Either of the triterpenoids and their potential target mechanisms may play a key regulatory role in our OA model and contribute to the antiarthritic action of SNO.

Although the protective potential of oral SNO supplementation differs from that of IAHA in many aspects (route, dose frequency, and mechanism of action). But the combination of both therapeutic models, like IA corticosteroid/anti-inflammatory drugs combined with IAHA, can lead to significant improvement of the clinical outcome of either agent alone [30]; this however only provides short duration, and is not feasible for persisting injection. Therefore, we sought to determine the role of oral SNO as an adjuvant in combination with IAHA injections and compare the effectiveness in the OA rat model. In this study, we compared the differences in protective potential between SNO and IAHA in OA rats, and the hypothetical synergetic effect of SNO with IAHA on the prevention of OA progression was also examined.

## 2. Materials and Methods

### 2.1. ACLT + MMx-Induced OA Animal Model

A rat model of surgically-induced OA was proceeded as described previously [20,21], and all animal care and experimental protocols complied with institutional and international standards (Principles of Laboratory Animal Care, National Institutes of Health) and were approved (Institutional Animal Care and Use Committee [IACUC] no. 107-030) by the IACUC of Cathay General Hospital (Taipei, Taiwan). Adult male Wistar rats were purchased from BioLASCO Taiwan Co., Ltd. (Yilan, Taiwan) and housed in Cathy Medical Research center with free access to the standard diet and water with a 12-h light/dark cycle at a temperature of 22 ± 2 °C and 55% humidity.

All ACLT + MMx surgeries were performed on the right knee of the rats by a single research specialist. Briefly, male Wistar rats (330–350 g) were anesthetized in an induction chamber using 5% isoflurane and then maintained with 2% isoflurane via a custom-made facemask. The right knee joint skin was shaved, and sterilized with povidone-iodine solution. An incision was made in the medial aspect of the joint capsule; the anterior cruciate ligament was transected using a scalpel, and the medial meniscus was removed completely using a tenotomy scissor. Following surgery, the joint was irrigated with normal saline; the joint capsule was sutured with 4–0 Vicryl, and 4–0 monofilament nylon was used for skin closure. Next, the wound area was sterilized, and cefazolin (100 mg/kg/day) was administered intramuscularly for 3 days to prevent infection. For the sham-operated rats, the same procedure was repeated, but neither ACLT nor removal of the medial meniscus was performed.

### 2.2. Experimental Design

**Figure 1:**
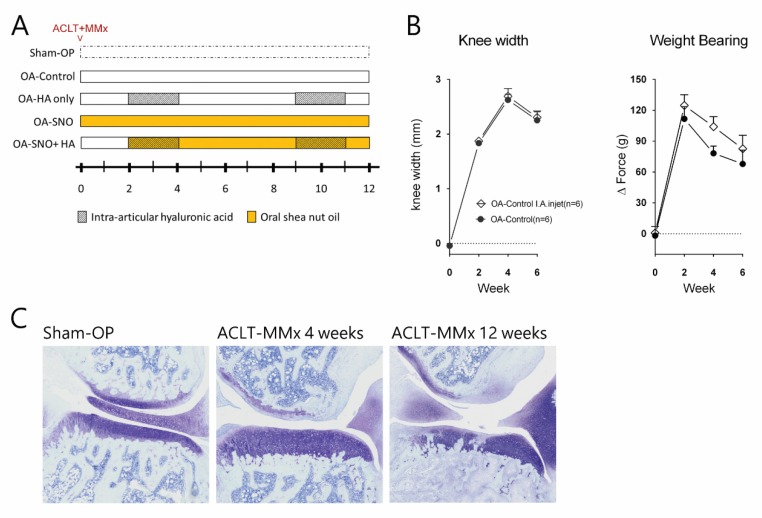
(**A**) The graphic scheme of study. (**B**) The knee width/weight-bearing changes of OA (osteoarthritis) rats with 3 IA (intra-articular) injection (weekly) of 50 μL of saline, compared to non-injected control. (**C**) The representative section of histological change of ACLT + MMx (anterior cruciate ligament transection plus medial meniscectomy)-induced OA from 4 weeks to 12 weeks post-surgery as compared to sham-OP (sham operated) rats.

As shown in Figure 1A, ACLT + MMx (*n* = 48) or sham surgery (Sham-OP, *n* = 9) was performed at week 0. Body weights, widths of the knee joints, and weight-bearing symmetry were measured before the surgery as the baseline. After the surgery, ACLT + MMx rats were assigned as the nontreated control (OA-control, *n* = 12), the group treated with SNO (223.2 mg/kg, OA-SNO, *n* = 12, administered oral SNO daily after the surgery, the effective dose was derived from our SNO dose-dependent animal report [20]), the group treated with HA (50 µL per joint/week, OA-HA, *n* = 12, received IA injection of HA weekly at weeks 2–4 and 9–11) and a group with combined treatment, HA plus SNO (OA-SNOHA, IA injection of HA(50 µL per joint/week) at weeks 2–4 and 9–11 plus the daily oral SNO(223.2 mg/kg) beginning from the 2nd week). The SNO concentrate provided by Universal Integrated Corp. (Taipei, Taiwan) was administered by oral gavage with the aid of isoflurane anesthesia, and the HA (Seikagaku Corporation, Ibaraki, Japan) was injected into the OA knee joint with a 25 G-needle syringe. As an injection control, 50 μL of saline solution was injected to OA-control (*n* = 6) at week 2–4 (3 weekly injections), and it showed no statistical difference in both knee width and weight-bearing test from non-injected OA-control rats (*n* = 6) (Figure 1B). The 4th week and 12th week knee joint section confirmed the progressive deterioration of OA joint with a reactive chondrocytes hypertrophy, and increasing cartilage erosion accompanied by chondrocytes loss (Figure 1C).

### 2.3. Knee Width and Weight-Bearing Test

The width of the knee joint was measured using a steel caliper (resolution 0.01 mm, E-Base Measuring Tools Co., Taipei, Taiwan) biweekly after the surgery, and the width of the contralateral knee served as the naïve control. The data are expressed as the Δ knee width (mm); the value was derived from the OA rats (knee width difference of the operated knee and naïve knee) minus the mean value of the sham-OP rats (knee width difference of the operated knee and naïve knee) and was determined as the actual joint swelling induced by ACLT + MMx.

Hind paw static weight-bearing was measured using an incapacitance tester (Linton Instrumentation, Norfolk, UK) to detect OA-induced changes in postural equilibrium every two weeks. The rats were placed on their hind paws in a box containing an inclined plane (65° from horizontal) that was placed above the apparatus. After a brief accommodation period, the weight that the animals applied to each hind limb was measured independently by the apparatus. Five measurements were taken and averaged for each rat. The data are expressed as the difference between the weight applied to the naïve hind limb and the weight applied to the operated hind limb (Δ Force, g); the change in the weight distribution between the naïve and operated hind limb represents the OA pain of the rats [31,32].

### 2.4. Histopathological Examination of Knee Joint

All rats were sacrificed via exsanguination under deep anesthesia on the 12th week post-surgery. The OA knee joints were removed and fixed in 10% formalin for 2 days, followed by a decalcifying solution based on EDTA disodium (12.5%, pH 7.0) for 4 weeks. After decalcification, the joints were embedded in paraffin blocks, and histological coronal sections (5 μm-thick serial section, slides interval: 200 μm) were obtained. Toluidine blue/fast green staining was used to examine morphological changes and the stained sections were digitalized using a Slide Scanner ZEISS Axio Scan Z1 image system (Jena, Germany) and ZEN lite 2.6 (blue edition). The severity of articular cartilage damage on medial tibial plateau was evaluated using the modified Osteoarthritis Research Society International (OARSI) scoring system [33]. The cartilage matrix loss width, tibia cartilage degeneration score, total and significant cartilage degeneration widths, and zonal depth ratio of the lesions and synovial reaction were evaluated.

### 2.5. Metabolic Profile of Blood Biochemistry Assays

The OA-SNO and OA-control rats fasted for 12 h before the blood sample withdrawal; blood samples were taken from the rat tail vein every 4 weeks post-ACLT + MMx surgery. The blood samples were centrifuged (8000 × g for 5 min) to separate sera and stored in a −80 °C freezer prior to analysis. The serological levels of uric acid, glucose, total cholesterol (T-CHO), high-density lipoprotein (HDL), and triglyceride (TG) were measured using the FUJI DRI-CHEM 4000i analyzer (FUJIFILM Corporation, Tokyo, Japan) at the Taiwan Mouse Clinic (Academia Sinica, Taipei, Taiwan).

### 2.6. Statistical Analysis

The data are expressed as the mean ± SEM. All graphical representations and statistical calculations were aided by GraphPad Prism version 6.01 and Microsoft Excel. The Shapiro–Wilk test was used to check the normal distribution of data. Two-way ANOVA, Tukey’s multiple comparisons test, Sidak’s multiple comparisons test, and Student’s t-test were used to analyze the data.

## 3. Results

### 3.1. SNO, but not HA, Attenuates Knee Joint Pain and Inflammatory Swelling in ACLT + MMx-Induced OA Rats

**Figure 2:**
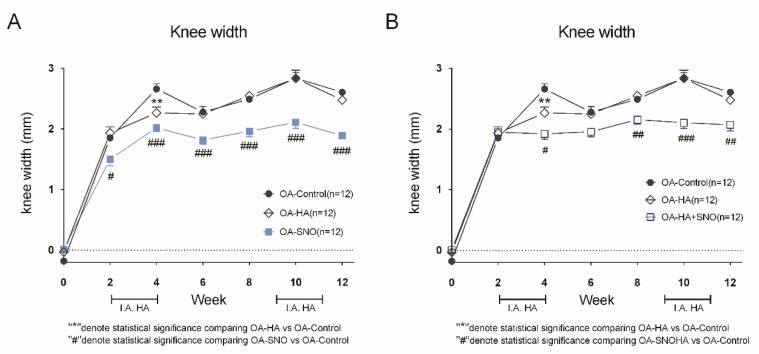
Joint swelling measurement of the OA rats. ACLT + MMx was performed to induce knee OA at week 0, and different treatments were given according to the experimental design. (**A**) Biweekly knee width measurements of the OA-control, OA-HA (hyaluronic acid), and OA-SNO (shea nut oil) rats. (**B**) Biweekly knee width measurements of the OA-control, OA-HA and OA-SNOHA (hyaluronic acid + shea nut oil) rats. The data are presented as the Δ knee width (mm), and the values are expressed as the mean ± SEM. Two-way ANOVA and Sidaks’s multiple comparisons test were used to analyze the data. # *p* < 0.05, ##/** *p* < 0.01, ### *p* < 0.001.

Immediately after ACLT + MMx surgery, rats were assigned to different treatment groups as described in Figure 1A. Following the surgery, in the OA-control group, we found that ACLT + MMx induced a constant and gradually increasing knee width as a result of progressive knee joint inflammation. In contrast, a preventive and long-term oral administration of SNO reduced the OA-induced knee joint swelling at 2 weeks post-treatment and eventually achieved a difference of approximately 27.5% (OA-SNO: 1.89 ± 0.06 vs. OA-control: 2.61 ± 0.05 mm) at week 12. However, such knee reduction was not evident in the HA-treated group (OA-HA: 2.48 ± 0.07 mm vs. OA-control: 2.61 ± 0.05 mm) (Figure 2A). With an oral SNO (daily) added to the HA treatment as an adjuvant supplement since the 2nd week, we started to observe the reduction in knee width from 4th to 12th weeks post-surgery in OA-SNOHA rats, (Figure 2B). At the end, we found a 20.6% reduction in the knee width compared to that of the OA-control group (OA-SNOHA: 2.07 ± 0.10 vs. OA-control: 2.61 ± 0.05 mm).

**Figure 3:**
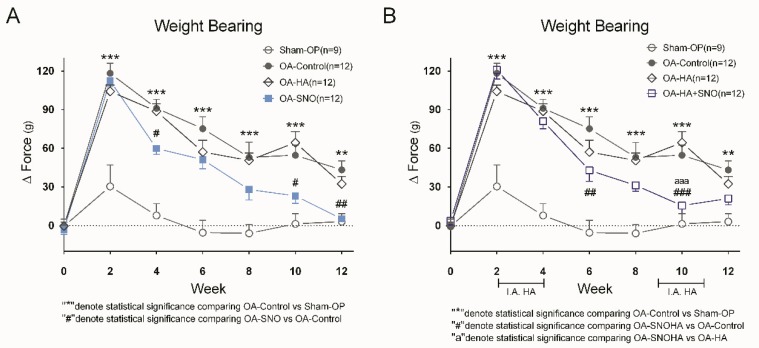
Weight bearing test of the OA rats. ACLT + MMx was performed to induce knee OA at week 0, and different treatments were given according to the experimental design. (**A**) Biweekly weight-bearing measurements of the sham-OP, OA-control, OA-HA, and OA-SNO rats. (**B**) Biweekly weight-bearing measurements of the sham-OP, OA-control, OA-HA, and OA-SNOHA rats. The data are presented as the Δ Force (g) and expressed as the mean ± SEM and two-way ANOVA, and Sidaks’s multiple comparisons test were used to analyze the data. # *p* < 0.05, ##/** *p* < 0.01, aaa/###/*** *p* < 0.001.

We used the weight-bearing test to assess pain behavior during OA progression (Figure 3A). Similar to our previous report, we found that ACLT + MMx-induced OA elicited a constant change in weight-bearing asymmetry compared to that of the sham-OP group, which only presented acute pain in the first few weeks as a result of the surgical procedure. The preventive and daily supplementation of SNO reduced the pain behavior up to 87.9% compared to that of the untreated OA-control group (OA-SNO: 5.22 ± 2.37 g vs. OA-control: 43.21 ± 6.93 g). However, HA alone did not yield any significant reduction in pain during our biweekly weight-bearing measurements; at the end, HA monotherapy yielded only a 25.3% reduction in treated rats compared to OA-control rats (OA-HA: 32.33 ± 5.90 g vs. OA-control: 43.21 ± 6.93 g). When the two treatments were given together since the 2nd week (Figure 3B), the OA-SNOHA rats showed a maximum reduction in pain (71.6%) at the 10th week as compared to O-control (OA-SNOHA: 15.51 ± 6.13 g vs. OA-control: 54.58 ± 7.90 g), and a 75.9% reduction when compared to the rats treated with OA-HA alone (OA-SNOHA: 15.51 ± 6.13 g vs. OA-HA: 64.37 ± 8.67 g).

**Figure 4:**
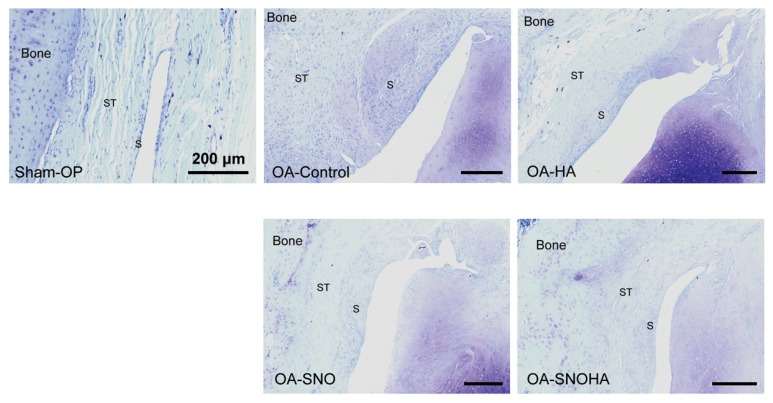
Synovium reaction of the OA joint. Representative sections of the medial femoral condyle joint capsule of the knee joints from the sham-OP, OA-control, OA-HA, OA-SNO, and OA-SNOHA rats at week 12 post-surgery were shown. ST = subsynovial tissue; S = synovial lining cells. Scale bar = 200 μm.

Next, we evaluated the synovial reaction as a microscopic sign of internal inflammation of the joint. In the sham-operated joint, we found a single layer of synovial lining cells without proliferation of the subsynovial tissue or vascular changes (Figure 4, sham-OP). As a result of the ACLT + MMx, the OA-control joint showed an increased thickness of both synovial lining cells, and the subsynovial tissue contained extensive extracellular matrix. Along with hypervascularity of the subsynovial area, this result suggested a chronic synovial reaction as result of surgically-induced OA (Figure 4, OA-control). Upon IAHA treatment (Figure 4, OA-HA), the histological findings showed a relative reduction in the synovial reaction compared to that of the OA-control joint, which suggested attenuation of OA with the lubricating action of IAHA. Moreover, with the addition of SNO supplementation to IAHA (Figure 4, OA-SNOHA) or oral SNO alone (Figure 4, OA-SNO), the two modalities of treatment offered better anti-inflammatory protection, as shown by the amelioration of the synovial reaction with less synovial proliferation and subsynovial thickness.

### 3.2. SNO, HA Alone, and HA Plus SNO, Offered Significant Improvement in Cartilage Integrity in Knee OA Rats

**Figure 5:**
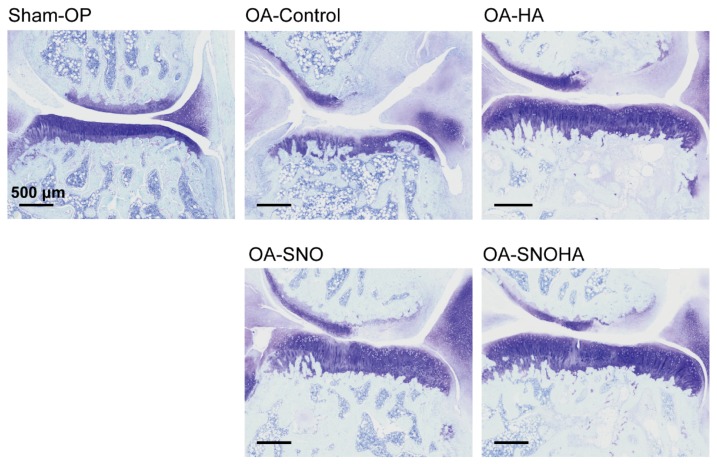
Representative section of medial tibial cartilage from each treatment (OA-HA, OA-SNO, and OA-SNOHA) and control (sham-OP, OA-control) were shown. Scale bar = 500 μm.

The cartilage deterioration was evaluated at 12th week post-surgery using the OARSI score system as shown in Table 1 and Figure 5. It is clearly noted that ACLT + MMx (OA-control) caused significant extensive matrix loss and a deformed cartilage surface, while the sham-OP cartilage showed a thin and smooth surface and preserved cartilage integrity. The quantitative data of the OA-HA, OA-SNO, and OA-SNOHA rats, showed a significant attenuation of cartilage matrix loss (specially in surface 0% and mild-depth 50% level) compared to that of the OA-control rats.

The cartilage degeneration score showed that ACLT + MMx induced a total lesion score of 6.208 ± 0.408, with the inside zone (2.708 ± 0.195) being the most affected region. The treatment with IAHA injection or combined SNO showed a significantly lower total degeneration score (5.000 ± 0.371 and 4.791 ± 0.307) than the OA-control (6.208 ± 0.408). In addition, we found that those rescues were observed primary in the inside zone, where all treatments (HA, SNO, and SNOHA) showed significant protective effects (1.833 ± 0.214, 1.833 ± 0.177, and 1.583 ± 0.133) compared to OA-control (2.708 ± 0.195).

Next, we quantified the extension (measured in width, mm) of the cartilage degeneration. The parameters are further subclassed as total (any type of degenerative change) or significant (seriously compromised, 50% of chondrocytes are absent or necrotic) and as the zonal depth ratio. Both OA-HA and combined treatment OA-SNOHA led to a significant attenuation of the 3 parameters, while OA-SNO more specifically decreased the significant cartilage degeneration width and zonal depth ratio. In summary, the oral SNO treatment alone significantly improved 5 of the 10 parameters we measured, while both IAHA and SNOHA treatment yielded significant ameliorations of 7 out of 10 parameters.

### 3.3. Metabolic Profiling of OA Rats Receiving Daily Oral Supplementation of SNO

**Figure 6:**
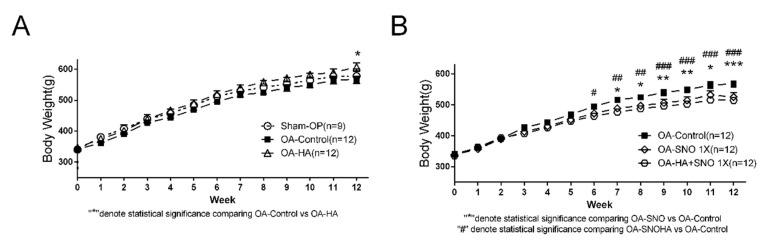

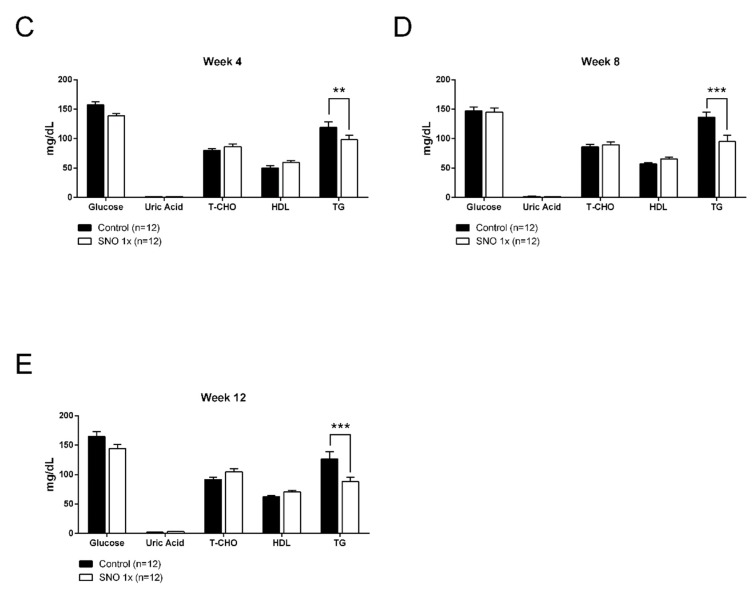
Body weight changes OA-control vs. OA-HA (**A**), or OA-control vs. OA-SNO and SNOHA (**B**). Metabolic profile of OA-control vs. OA-SNO rats (glucose, uric acid, total cholesterol, high-density lipoprotein (HDL), and triglyceride (TG)) at the 4th week (**C**), 8th week (**D**), and 12th week (**E**) after ACLT + MMx surgery. Values are expressed as the mean ± SEM, and two-way ANOVA and Tukey/Sidaks’s multiple comparisons test were used to analyze the data. #/* *p* < 0.05, ##/** *p* < 0.01, ##/*** *p* < 0.001.

Firstly, we found no difference of body weight between sham-OP and OA-control, but a significantly, but minor increase of body weight was detected in the OA-HA group at the 12th week (Figure 1A). On the other hand, we found gradual and minor reduction of body weight in OA rats supplemented with oral SNO, including those receiving IAHA at the same time (Figure 1B). We previously demonstrated that long-term oral SNO supplementation in OA rats decreased body weight and blood TG level without altering the blood aspartate transaminase (AST), blood urea nitrogen (BUN), and cholesterol levels [21]. Here, we compared the full metabolic profile at the 4th, 8th, and 12th weeks. Similarly, we found no alteration of uric acid, total cholesterol, and HDL at any of the time-points (Figure 6C–E). Consistent with our previous report, a significant reduction in TG levels was observed as early as the 4th week (118.8 mg/dL vs. 98.08 mg/dL) and remained reduced until the 12th week (126.7 mg/dL vs. 88.25 mg/dL) (Figure 6C–E).

## 4. Discussion

In the current study, we found that both daily oral SNO supplementation and 6 weekly doses of IAHA alone are sufficient to attenuate post-traumatic OA-induced cartilage deterioration. In terms of the knee joint swelling and pain assessment, we found a marked difference between the two modalities of treatment, in which the long-term daily oral SNO supplement resolved better reduction in inflammatory signs/symptoms of the knee as well as the synovial reaction in the joint cavity than IAHA alone. The combination of both treatments demonstrated an additive effect, the SNO + IAHA OA rats showed the best histological scores, and at 10 weeks of oral SNO supplement provided additional anti-inflammatory and antinociceptive effects on visco-lubricative IAHA chondro-protection. Moreover, long-term oral SNO supplementation caused no alteration in metabolic profiles, such as serological uric acid, total cholesterol, and HDL levels, compared to those of control OA rats. Surprisingly, there was a minor reduction in fasting blood glucose and significantly reduced TG levels in the SNO-treated rats.

It is worth noting, a reduction of body weight was also observed in SNO and SNOHA treated rats. This similar finding was reported recently in obese rats [29]. The anti-obesity effect of SNO could be the result of reduction of OA pain, or vice versa. Given the fact that the clinical and pathogenic correlation between OA and metabolic disorder has been extensively reviewed [34], thus the management of body weight is strongly recommended by OARSI, American Academy of Orthopaedic Surgeons (AAOS), and the American College of Rheumatology [35,36,37]. Clinical studies on weight loss and preclinical studies targeting metabolic abnormalities in OA are an area of research interest and have achieved important improvement in OA progression [38]. In rat model of type 2 diabetes mellitus, Onur et al. demonstrated the metabolic disease itself contributes to the onset and progression of knee osteoarthritis [39]. Mooney et al. showed that surgically-induced OA mice fed a high-fat diet presented not only higher fasting glucose levels and body weights compared to those of lean OA mice, but also had worse OARSI histological scores and less cartilage thickness [40]. Moreover, a recent report also demonstrated that cartilage deterioration was sustained even after the high-fat diet was withdrawn from the OA mice, and the blood glucose and body weight were restored to the levels in normal diet mice [41]. These findings suggested that an increased weight load is not the sole cause of the severity of OA progression; instead, the lipid/glucose metabolic pathways could also jeopardize cartilage integrity and synthesis.

Moreover, an emerging concept of gut–joint axis has associated the gut dysbiosis (perturbation of gut microbiota (GM) biodiversity and function), and the leaky gut syndrome with the joint disease progression [42,43]. In both human and rodent model, an increase in serum level of the pro-inflammatory marker and bacterial metabolites were associated with OA severity [44,45]. This chronic low-grade inflammation as result of dysbiosis explains a new OA phenotype, indicated as the metabolic OA [46]. In fact, long-term diet or prebiotic supplements have shown to shift GM colony with the improvement of cartilage integrity [47]. The diary ingestion of high triterpenes SNO could have potential modulation on the GM diversity, which may associate with both metabolic change and cartilage protection. Future evaluation of GM colonies under long-term oral SNO remains to be explored.

On the other hand, in vitro evidence shows that a mixture of triterpenes (α, β-amyrin) significantly reduced lipid droplet formation via suppression of PPARγ and C/EBPα expression, while enhancing the translocation of glucose transporter GLUT4 onto the plasma membrane of 3T3-L1 cells [48]. Furthermore, reduction in blood glucose, total cholesterol, and TG levels were also observed in streptozotocin-induced diabetic mice treated with an α, β-amyrin mixture [49]. Moreover, triterpene as lupeol was also found to have a hypolipidemic effect (decreased total cholesterol, TG, and phospholipids) in rats fed a high cholesterol diet [50]. The effect of SNO (with a high concentrate of triterpenes) on lipid metabolism may be connected to the molecular mechanism of its chondro-protective effect.

As previously shown, the predominant fatty acids in femoral head cartilage are palmitic (16:0), oleic (18:1), and linoleic (18:2) acids [51]. In the animal model of OA, those fatty acids were significantly reduced in mice after destabilization of the medial meniscus [52]. Nonetheless, oleic acid exposure downregulates the expression of MMP-1 and COX-2 in TNF-α stimulated human chondrocytes culture while linoleic acid increased PGE2 production [53]. These results suggest that local fatty acid concentrations could be results of OA and also contribute to OA progression. The high proportion of oleic acid of SNO could be one of the chondro-protection factors.

The interval and multiple doses of IAHA showed a significant chondro-protective effect in the treated group compared with the OA-control in our ACLT + MMx injured OA rats. Surprisingly, we observed neither an antinociceptive effect nor a reduction in inflammatory signs, as demonstrated by the weight-bearing asymmetry and knee swelling tests. In fact, several animal studies on OA pain reported similar findings on the HA effect. Ikeuchi et al. employed a monoiodoacetate (MIA)-induced OA pain model and found no significant difference in weight-bearing asymmetry in HA-treated rats [54]. Boettger et al. also demonstrated in a rat bradykinin/PGE2 pain model that HA lost its antinociceptive efficacy (shown as weight-bearing asymmetry) from day 7 after injection [55]. Recently, IAHA was found unable to reduce ankle swelling in MIA-induced ankle OA [56], which is similar to the knee width exam in our ACLT + MMx OA model. However, the limited time of follow-up and the small sample size are the two major limitations of this study. An evaluation with an extended follow-up observation will further elucidate the long-term effect of IAHA alone or in combination with oral SNO for the treatment of chronic osteoarthritis.

## 5. Conclusions

Although pharmacological treatments of OA are rapid and effective for symptomatic relief in regular clinical practice, the long-term use is restricted by the associated adverse effects. IAHA has been conditionally recommended for long-term treatment of knee OA with a favorable safety profile over repeated IA corticosteroid. At and beyond 12 weeks of treatment, it may have the beneficial effects on pain [57]. Nutraceuticals are safe candidates for long-term supplementation to provide persistent effect as treatment adjuvant [58]. In fact, many nutraceutical products have been extensively used for OA pain and their active compounds were identified for potential drug development [59]. In conclusion, the evidence and safety profiles observed in these surgically-induced OA rats suggest that long-term oral SNO supplement can be used as an effective adjuvant for IAHA treatment to enhance the symptomatic relief and delay the disease progression in clinical practice.

## Figures and Tables

**Table 1:** Histological scoring of OA knee joint.

	Sham-OP (*n* = 7)	OA-Control (*n* = 12)	OA-HA (*n* = 12)	OA-SNO (*n* = 12)	OA-SNOHA (*n* = 12)
Cartilage matrix loss 0% (mm)	0 ***	2.292 ± 0.074	1.951 ± 0.087 **	1.864 ± 0.107 **	1.865 ± 0.060 ***
Cartilage matrix loss 50% (mm)	0 ***	0.792 ± 0.144	0.386 ± 0.102 *	0.342 ± 0.103 *	0.272 ± 0.116 **
Cartilage matrix loss 100% (mm)	0 **	0.372 ± 0.093	0.183 ± 0.076	0.217 ± 0.075	0.160 ± 0.061
Medial Tibia Cartilage Degeneration Score	0 ***	6.208 ± 0.408	5.000 ± 0.371 *	5.750 ± 0.439	4.791 ± 0.307 **
└ Outside zone	0 ***	1.625 ± 0.334	1.458 ± 0.289	1.875 ± 0.326	1.375 ± 0.287
└ Middle zone	0 ***	1.875 ± 0.163	1.708 ± 0.185	2.042 ± 0.153	1.833 ± 0.130
└ Inside zone	0 ***	2.708 ± 0.195	1.833 ± 0.214 **	1.833 ± 0.177 **	1.583 ± 0.133 ***
Total cartilage degeneration width (mm)	0 ***	2.475 ± 0.073	2.213 ± 0.070 *	2.335 ± 0.073	2.215 ± 0.058 **
Significant cartilage degeneration width (mm)	0 ***	0.947 ± 0.111	0.612 ± 0.110 *	0.528 ± 0.127 *	0.368 ± 0.117 ***
Zonal depth ratio of lesions	0 ***	0.469 ± 0.039	0.358 ± 0.029 *	0.331 ± 0.024 **	0.313 ± 0.020 ***

All operated knee joints were collected at 12 weeks post-surgery and were processed with toluidine/fast green staining for evaluation using OARSI’s parameters. The bold text shows the value with statistical significance. Asterisk denote the statistical examination of each group in comparison with OA-control using Student’s *t*-test. * *p* < 0.05, ** *p* < 0.01, *** *p* < 0.001
